# Impact of the Presence of Chronic Total Occlusions on the Survival of Patients Treated with Coronary Artery Bypass Grafting

**DOI:** 10.3390/jcdd12070243

**Published:** 2025-06-25

**Authors:** Albi Fagu, Joseph Kletzer, Franziska Marie Ernst, Laurin Micek, Stoyan Kondov, Maximilian Kreibich, Clarence Pingpoh, Matthias Siepe, Martin Czerny, Tim Berger

**Affiliations:** 1 Department of Cardiovascular Surgery, University Heart Center Freiburg—Bad Krozingen, University Medical Center Freiburg, 79106 Freiburg, Germany; joseph.kletzer@uniklinik-freiburg.de (J.K.); franziskamarie.ernst@gmail.com (F.M.E.); laurin.micek@uniklinik-freiburg.de (L.M.); stoyan.kondov@uniklinik-freiburg.de (S.K.); maximilian.kreibich@uniklinik-freiburg.de (M.K.); clarence.pingpoh@uniklinik-freiburg.de (C.P.); martin.czerny@uniklinik-freiburg.de (M.C.); tim.berger@uniklinik-freiburg.de (T.B.); 2 Faculty of Medicine, University of Freiburg, 79110 Freiburg, Germany; 3 Department of Morphology, Faculty of Medicine, University of Medicine Tirana, 1029 Tirana, Albania; 4 Department of Cardiac Surgery, University Hospital Bern, University of Bern, 3012 Bern, Switzerland; matthias.siepe@insel.ch

**Keywords:** coronary artery bypass grafting (CABG), chronic total occlusion (CTO), coronary revascularization

## Abstract

Although chronic total occlusions (CTO) are a common finding in patients treated with coronary artery bypass grafting (CABG), it is still not clear how their presence impacts the long-term outcomes achieved with surgery. We aimed to investigate the impact of CTO on the long-term results of patients with coronary artery disease who underwent CABG. Patients from 2005 to 2023 operated on at the University Hospital Freiburg-Bad Krozingen were analyzed. The primary outcome was all-cause mortality after 3-, 5-, and 10 years. The secondary outcome was the need for coronary reintervention in the follow-up period. Propensity score matching and multivariable Cox regression were performed, and Kaplan–Meier curves were used to graphically display the outcomes for the two groups. Of the 3424 patients included in the analysis, 1784 (52%) were categorized as CTO and 1640 (48%) were categorized as no-CTO. After propensity scoring, 1232 pairs were successfully matched. The 3-, 5-, and 10-year all-cause mortality was significantly higher in patients with CTO (*p* = 0.028; *p* < 0.001; *p* < 0.001). The need for coronary reintervention after 3-, 5-, and 10 years was comparable in both groups. In addition, multivariable Cox Regression showed that CTO presence (HR 1.220, 95% CI 1.047–1.420, *p* = 0.010) was an independent predictor of 10-year mortality.

## 1. Introduction

Chronic total occlusions (CTOs) are a common finding in patients with advanced coronary artery disease (CAD), with a prevalence that differs considerably between patients treated with percutaneous coronary intervention (PCI) and coronary artery bypass grafting (CABG) [1,2]. Occlusions occur gradually and enable coronary collateralization. Therefore, they are consistently found in patients with chronic coronary syndromes, or in symptomatic patients due to other non-occlusive lesions [3]. The collateralization of the CTO helps limit myocardial injury, and only a minority present with a severely reduced left ventricular ejection fraction [1,4].

A CTO represents the most advanced form of atherosclerotic involvement of the coronaries and is an indicator of CAD severity [5,6]. These patients can differ considerably from other CAD patients. They are generally older and have a higher burden of cardiovascular risk factors. The majority have multivessel disease, and in 7–10%, the left main artery is involved, generally with concomitant severe calcification of the coronary arteries [4,7]. The higher atherosclerotic burden and local endothelial dysfunction consequently influence the progression from advanced CAD to CTO [8].

Different studies including patients treated by PCI have confirmed the negative impact of CTOs on outcomes in patients with stable angina, acute coronary syndrome, or ischemic heart failure [9,10]. The extensive revascularization and collateralization achieved by CABG, particularly in patients with advanced CAD, should, at least to some extent, reduce the impact of CTO on patient outcomes. On the other side, myocardial viability, CTO collateralization, and grade of atherosclerotic involvement of the coronaries may also influence these outcomes. Studies evaluating the effect of CTO on the outcomes of patients treated with bypass surgery are scarce and include a small number of patients [11,12].

The aim of this study was to evaluate the prognostic impact of the presence of CTOs on long-term outcomes in patients treated with CABG.

## 2. Patients and Methods

### 2.1. Study Population

This was a retrospective study of patients who underwent CABG at the University Heart Center Freiburg—Bad Krozingen between 2005 and 2023. Included were all patients who received isolated CABG for elective or emergent indication, with or without preoperative inotropic or mechanic support. Exclusion criteria were previous cardiac operations and concomitant valvular or proximal thoracic aortic surgery.

### 2.2. Data Collection

Our dedicated institutional CABG database was used to retrieve the data. Patients were divided into CTO and no-CTO groups. Patient baseline characteristics, disease-specific variables, comorbid conditions, procedural variables, and laboratory data were extracted from medical records. The surgical protocols were checked for intraoperative variables. Survival was determined through manual querying of the federal resident registration office.

### 2.3. Definition of Parameters

A total occlusion (interrupted antegrade flow) was considered chronic when known or assumed to be ≥3 months old [13]. Small, occluded vessels (≤1.5 mm) were considered not revascularizable and these patients were included in the no-CTO group. Reintervention was defined as a repeat surgical or cardiological procedure for ischemic heart disease. Complete revascularization was defined according to the criteria of the ROMA study [14]. Mortality was defined as death from any cause. Stroke was defined as clinical evidence of neurological deficit with radiological evidence of ischemia or bleeding. Deep sternal wound infection was defined as infection of the sternum with bacterial evidence and the need for surgical and anti-infective therapy.

### 2.4. Definition of Primary and Secondary Outcomes

The primary outcome was defined as all-cause mortality after 3, 5, and 10 years.

The secondary outcome was defined as a repeat procedure on the coronary arteries via surgery or PCI after 3, 5, and 10 years.

### 2.5. Statistical Analysis

Statistical analysis was performed using R Studio software (version 4.3.3) (RStudio: Integrated Development for R. RStudio, Inc., Boston, MA, USA). The Shapiro–Wilks test was used to assess the distribution of continuous data. Normally distributed data were expressed as ± standard deviation and compared using the unpaired *t*-test. Otherwise, the non-parametric Mann–Whitney-U test was used, and data were expressed as the median and interquartile range. For categorical data, the Chi-square test was used. A *p*-value < 0.05 was deemed statistically significant.

To address confounders and strive for pseudo-randomization, we performed propensity score matching before analyzing the data. To address missing data, we performed multiple imputations using the mice package (Multivariate Imputation by Chained Equation) using the CART algorithm. Matching was then performed using 1:1 optimal pair matching with propensity score distances estimated with logistic regression. Detailed descriptions of the statistical method, density plots, absolute standardized mean difference plots, and propensity score distribution plots can be found in the Supplemental Materials. To illustrate mortality and coronary reintervention, Kaplan–Meier plots were used after matching. Additionally, multivariate, multivariable Cox regression was used to estimate hazard ratios for the outcomes over 10 years of follow-up.

## 3. Results

### 3.1. Patient Characteristics

A total of 3424 patients underwent surgical revascularization within the study period. Among these, 1784 (52%) had at least one CTO. The cohort’s median age was 68.0 (62.0, 74.0) years, with patients from the no-CTO group being significantly older 68.0 (61.0, 74.0) years vs. 69.0 (63.0, 75.0) years; *p* < 0.001). The most common risk factors were hypertension (83%; 85% vs. 82%, *p* = 0.019), followed by dyslipidemia (80%; 84% vs. 76%, *p* < 0.001) and smoking (37%; 41% vs. 33% *p* < 0.001). Prior MI was present in 34% (35% vs. 32%, *p* = 0.029) of patients and 6.1% of them had a history of stroke. The majority of patients (99%) had multivessel disease, with patients from the CTO group more frequently having 3-vessel disease (95% vs. 87%). The left ventricular function was severely reduced in 88 (2.8%) patients (3.3% vs. 2.1%, *p* = 0.032). The median Euroscore II was 1.3 (0.9, 2.0) with a significant difference between the two groups (1.4 (0.9, 2.1) vs. 1.2 (0.9, 1.9) *p* = 0.004). Emergency surgery was necessary in 197 (8%) cases. After propensity score matching, 1232 pairs with comparable preoperative characteristics were formed. The preoperative characteristics and comorbidities after propensity matching are summarized in Table 1 (Characteristics and outcomes of the unmatched groups can be found in the Supplemental Materials).

### 3.2. Intraoperative Data

CTO distribution and revascularization rates are shown in Table 2. Successful revascularization was achieved in 92% of LAD-CTO, 85% of RCA-CTO, and 79% of LCX-CTO. On average, 3.1 (0.9) distal anastomoses were completed, with the CTO patients receiving significantly more anastomoses (3.2 (0.9) vs. 3.0 (0.8) *p* < 0.001). An anatomically complete revascularization was achieved in 2798 (91%) patients, with no significant differences between the two groups. The median CPB time (90 min (74, 107)) was significantly longer in the CTO group (91 min (78, 107) vs. 88 min (71, 106), *p* < 0.001), while the aortic cross-clamp time was similar (69 min (55, 84); 69 min (58, 83) vs. 68 min (53, 85)). After propensity score matching, only the number of anastomoses remained significantly different between the groups. The procedural characteristics are summarized in Table S2 (Supplemental Materials).

### 3.3. Early Clinical Outcomes

The overall 30-day mortality rate was similar between groups after matching (1.5% vs. 1.3%, *p* = 0.7). Rates of postoperative dialysis, stroke, and deep sternal infection were comparable. Postoperative atrial fibrillation occurred more frequently in the CTO group (20% vs. 17%, *p* = 0.020). The 30-day results are summarized in Table 3.

### 3.4. Follow-Up Data

The overall 3-, 5-, and 10-year all-cause mortality rates were 8.1% (n = 277), 12% (n = 417), and 23% (n = 778). The mortality was significantly higher in the CTO group (7.6% vs. 5.4%, *p* = 0.028; 12% vs. 8.1%, *p* < 0.001; 24% vs. 15%, *p* < 0.001). The rate of coronary reinterventions showed no significant difference after 3-, 5-, and 10-years (*p* > 0.9, *p* = 0.5, *p* = 0.2). The follow-up data are summarized in Table 4. After multivariable Cox Regression of the matched cohort, it was found that CTO presence (HR 1.220, 95% CI 1.047–1.420, *p* = 0.010), age (HR 1.055, 95% CI 1.044–1.066, *p* < 0.001), history of smoking (HR 1.488 95% CI 1.270–1.743, *p* < 0.001), and NYHA Class (HR 1.936 95% CI 1.051–3.567, *p* = 0.034) were independent predictors of 10-year mortality (Table 5).

**Figure 1:**
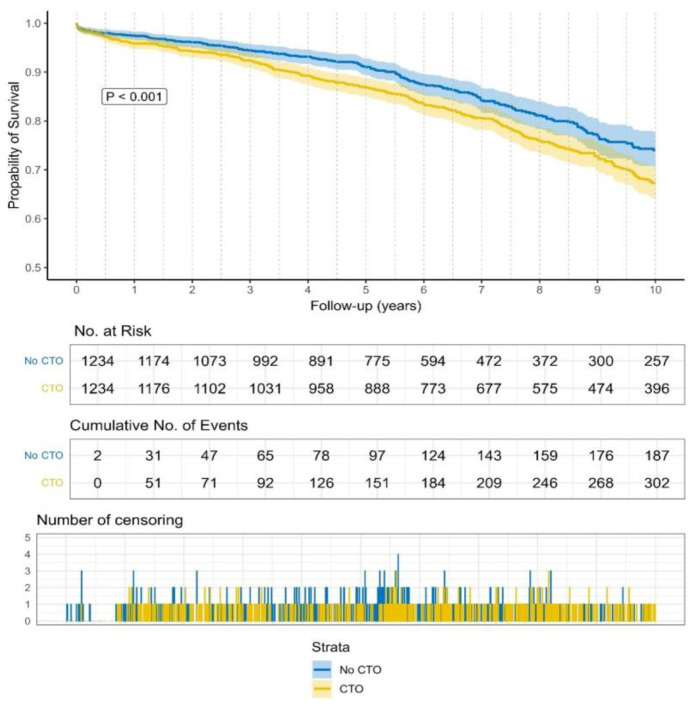
Probability of survival over 10 years.

**Figure 2:**
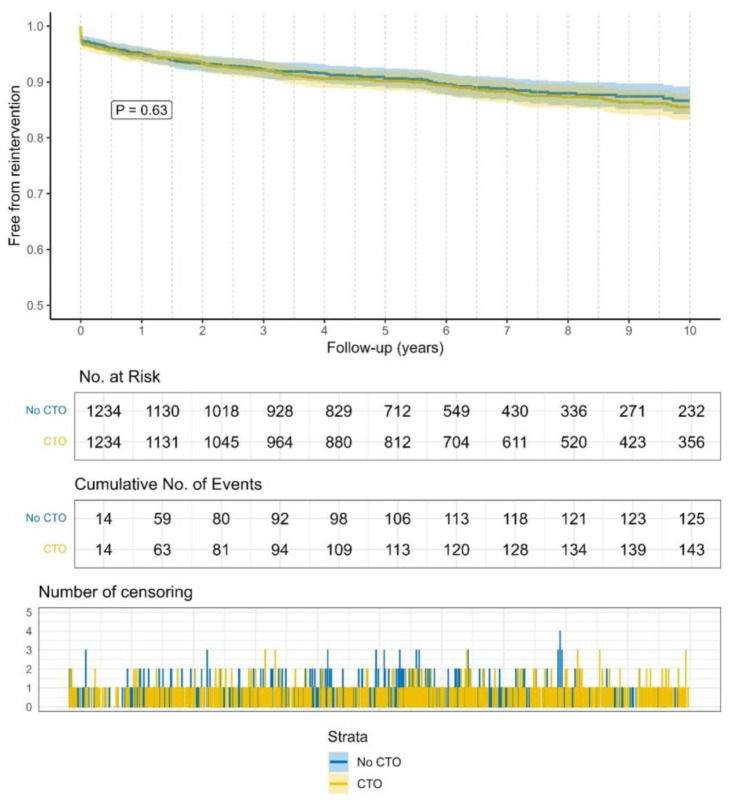
Freedom from reintervention over 10 years.

Kaplan–Meier curves showed a lower probability of survival (Log Rank *p*-value < 0.001) in CTO patients. The need for reintervention was comparable between the two groups (Log Rank *p* = 0.63). Kaplan–Meier curves are illustrated in Figure 1 and Figure 2.

## 4. Discussion

The three most essential findings of our comparison of CTO vs. no-CTO patients can be summarized as follows:

(I) The presence of CTO was a predictor of 3-, 5-, and 10-year mortality before and after statistical adjustments. However, this effect was weaker than other investigated factors.
(II) Coronary reintervention rates remained low, even after 10 years, indicating a durable treatment.
(III) The treatment of CTO with CABG revealed very favorable long-term outcomes.

The CTO patients in our unmatched cohort were younger than their no-CTO counterparts but had significantly more risk factors (Table S1, Supplemental Materials). The age difference between the groups may have been influenced by the selection of patients referred to CABG. Although there are no specific recommendations regarding the treatment of patients with CTO, the actual guidelines clearly identify CABG as the treatment of choice for complex CAD [15,16]. Different studies have confirmed that CTO patients have higher SYNTAX scores when compared with no-CTO counterparts [11,12]. In our database, this information is missing, but we think that CTO presence and SYNTAX scores influenced the outcomes in the same direction. In centers with a high level of expertise in complex PCI procedures, the group of patients referred for surgical revascularization is only a selected subset of the general CAD population. Compared to large CABG and PCI cohorts, our patients were older and had a higher cardiovascular risk profile, with a higher rate of patients with diabetes and peripheral arterial disease [7,17]. Different studies including patients treated with PCI have confirmed the negative impact of CTOs on outcomes in various subsets of patients [9,10]. Gold et al. and Tsao et al. reported that CTO presence is related to increased mortality, incidence of cardiovascular death, and myocardial infarction [5,6]. Moreover, the successful recanalization of CTO (PCI-CTO) has been shown to improve symptoms, quality of life, and ischemic burden, but the impact on preventing clinical events remains unclear [12,18,19,20]. Theoretically, the extensive revascularization and collateralization achieved by surgery should, at least to some extent, reduce the impact of CTOs on the outcomes of these patients, regardless of CTO revascularization. Complete surgical revascularization is normally not influenced by the presence of a CTO and can almost always be achieved in one procedure, without significantly prolonging the operation. The complexity of CAD and level of experience do not necessarily influence intraoperative times [21]. On the other hand, staged procedures and prolonged times are common during the more complex CTO-PCI. Previous works have already demonstrated that complete myocardial revascularization is associated with superior long-term outcomes [17]. Nevertheless, myocardial viability, CTO collateralization, and grade of atherosclerotic involvement of the arteries are additional factors that could influence these outcomes. The difference in mortality rates persisted even after adjustments for baseline characteristics, confirming that factors related to atherosclerosis and myocardial perfusion may have affected the results. We believe these factors may also influence the outcomes in a surgical population and probably impact graft patency rates. Angiographic studies may be needed to further elucidate the mechanisms explaining this difference. In addition, we cannot differentiate between cardiovascular and non-cardiovascular death, and cannot weigh their specific influence on all-cause mortality.

The need for coronary reintervention was relatively low and not significantly different between the two groups under comparison. Repeat revascularization rates were low, even when compared with CTO-PCI studies. The combination of multivessel disease and CTO may lead to lower rates of complete revascularization when treated with PCI [22]. The CTO subset of the SYNTAX trial showed significant differences between PCI-CTO and CABG-CTO groups regarding complete revascularization and successful CTO revascularization [7]. Lin et al. reported repeat revascularization rates of as high as 21.2% in their CTO-PCI group after 5 years [23]. In a meta-analysis of studies comparing outcomes in patients undergoing CABG or PCI for CTO, Kirov et al. reported that repeat revascularization was higher in the PCI group [24]. The fundamentally different technical and physiological approach in CTO revascularization with CABG and higher levels of complete revascularization can explain the lower repeat revascularization rates and the lack of difference between CTO and non-CTO patients. In fact, CABG addresses the distal part of the coronary arteries, and the revascularization technique is independent from the proximal lesion. Moreover, by avoiding the CTO, the risk of distal emboli resulting from “crushing” and “mobilizing” the atherosclerotic material/plaques is low in the case of CABG.

The overall all-cause mortality after 5 years was 12% (12% vs. 8.1% after matching) and 23% after 10 years (24% vs. 15% after matching), with a significant difference between CTO and no-CTO patients before and after adjustment. In a similar cohort, but with younger patients, Fefer et al. reported very similar 5-year results [11]. Roth et al., in a comparison between PCI-CTO and CABG-CTO, reported a 5-year mortality of 21% for a very similar cohort [12]. Comparisons with the literature for PCI-CTO can be difficult as a result of considerable differences in patient characteristics. In a recent publication, Gold et al. reported a 5-year mortality of 28.6% in 3597 patients who were treated with PCI. Of these, 922 (26%) had at least 1 CTO and only 31.2% had 3-vessel disease [5]. This is a considerable difference from our group of patients, with 3-vessel disease in 91% of cases. In general, patients treated with PCI-CTO are younger, have a lower risk profile, and less often have 3-vessel disease [5]. They constitute a preselected subgroup and, as expected, this also influences the long-term outcomes.

To the best of our knowledge, this is the largest surgical cohort to date to evaluate the impact of CTO in patients treated with CABG. It confirms the results of previous reports and the negative impact of CTO on the long-term results achieved with surgery.

## 5. Conclusions

This study demonstrates that the long-term results achieved by CABG in patients with advanced CAD and CTO are very favorable. CTO presence is an independent predictor of long-term mortality in this group of patients. The repeat revascularization rates were low, even after 10 years, and were not influenced by CTO.

## 6. Limitations

This is a retrospective study, with all limitations related to its design. The patients referred to CABG were a selected subgroup and probably do not represent all patients treated for CAD. Using cardiac mortality instead of all-cause mortality as the primary outcome could be more appropriate for this cohort. The more appropriate choice of cardiovascular mortality as the primary outcome measure instead of all-cause mortality is supported by the fact that at 10 years the competitive risk of death from non-cardiac causes is significant (i.e., cancer, as suggested by the independent prognostic value of the history of malignancy shown in Table 5). Postoperative angiographic data and patency rates are lacking and could provide valuable information. Furthermore, selection bias could be addressed through a prospective randomized study design.

## Figures and Tables

**Table 1:** Patient characteristics.

		CTO/No CTO (Matched)		
Characteristics	Overall, N = 3424 *	CTO, N = 1232 *	No CTO, N = 1232 *	*p*-Value **
		Matched	Matched	
Sex (female)	532 (15%)	172 (14%)	186 (15%)	0.4
Age (years)	68.0 (62.0, 74.0)	69.0 (63.0, 74.0)	69.0 (63.0, 74.0)	>0.9
BMI (kg/m^2^)	27.4 (25.1, 30.1)	27.7 (25.4, 30.6)	27.7 (25.4, 30.5)	0.5
Diabetes	1082 (32%)	390 (32%)	394 (32%)	0.9
Insulin dependent Diabetes	386 (10%)	125 (10%)	125 (10%)	>0.9
Dyslipidemia	2728 (80%)	1027 (83%)	1027 (83%)	>0.9
Hypertension	2844 (83%)	1053 (85%)	1046 (85%)	0.7
Arrhythmia	294 (8.6%)	103 (8.4%)	110 (8.9%)	0.6
Smoking	1172 (37%)	469 (38%)	436 (35%)	0.2
PAD	447 (15%)	167 (14%)	169 (14%)	>0.9
COPD	349 (10%)	122 (9.9%)	113 (9.2%)	0.5
History of MI	1149 (34%)	397 (32%)	390 (32%)	0.8
History of stroke	209 (6.1%)	80 (6.5%)	62 (5.0%)	0.12
Malignancy	60 (1.9%)	22 (1.8%)	23 (1.9%)	0.9
NYHA				0.4
1	341 (11%)	154 (13%)	164 (13%)	
2	1449 (47%)	595 (48%)	613 (50%)	
3	1213 (39%)	470 (38%)	448 (36%)	
4	81 (2.6%)	13 (1.1%)	7 (0.6%)	
Chronic renal failure	97 (2.9%)	18 (1.5%)	14 (1.1%)	0.5
EuroScore II	1.3 (0.9, 2.0)	1.2 (0.9, 1.8)	1.2 (0.9, 1.8)	0.2
Preop. LVEF < 30%	88 (2.8%)	5 (0.4%)	5 (0.4%)	>0.9
Significantly stenosed vessels				>0.9
1	24 (1%)	1 (<0.1%)	1 (<0.1%)	
2	283 (8%)	53 (4%)	53 (4%)	
3	3108 (91%)	1178 (96%)	1178 (96%)	
CSS Classification				0.002
CCS 0	258 (8.4%)	115 (9.3%)	90 (7.3%)	
CCS 1	114 (3.7%)	51 (4.1%)	40 (3.3%)	
CCS 2	816 (27%)	359 (29%)	314 (26%)	
CCS 3	1244 (41%)	514 (42%)	531 (43%)	
CCS 4	638 (21%)	192 (16%)	255 (21%)	

* Median (IQR); n (%); Mean (SD), ** Wilcoxon rank sum test; Pearson’s Chi-squared test; Fisher’s exact test; BMI, Body Mass Index; COPD, Chronic Obstructive Pulmonary Disease; LVEF, Left Ventricular Ejection Fraction; MI, Myocardial Infarction; NYHA, New York Heart Association, PAD, Peripheral Artery Disease.

**Table 2:** CTO distribution and revascularization rates.

CTO Distribution	Overall, N = 3424	CTO, N = 1232
		Matched
CTO LAD	439 (25%)	285 (23%)
CTO RCA	1174 (67%)	826 (67%)
CTO LCX	584 (33%)	403 (33%)
Revascularization of CTO LAD	408 (92%)	265 (93%)
Revascularization of CTO RCA	1001 (85%)	702 (85%)
Revascularization of CTO LCX	464 (79%)	315 (75%)

CTO, Chronic Total Occlusion; LAD, Left Anterior Descending; RCA, Right Coronary Artery; LCX, Left Circumflex Artery.

**Table 3:** Thirty-day outcomes.

		CTO/No CTO (Matched)		
Characteristic	Overall, N = 3424 *	CTO, N = 1232 *	No CTO, N = 1232 *	*p*-Value **
30-day mortality	59 (1.7%)	18 (1.5%)	16 (1.3%)	0.7
Coronary reintervention after 30 days	95 (2.8%)	37 (3.0%)	35 (2.9%)	0.8
Re-CABG after 30 days	4 (0.1%)	1 (<0.1%)	1 (<0.1%)	>0.9
PCI after 30 days	95 (2.8%)	37 (3.0%)	36 (2.9%)	>0.9
Hospital stay (days)	14.0 (11.0, 17.0)	14.0 (11.0, 17.0)	13.0 (11.0, 16.0)	<0.001
Length of ICU stay (days)	1.0 (0.9, 1.9)	0.9 (0.9, 1.8)	0.9 (0.8, 1.6)	0.10
Pacemaker implantation after 30 days	49 (1.4%)	18 (1.5%)	17 (1.4%)	0.9
Postoperative Dialysis	113 (3.3%)	26 (2.1%)	29 (2.4%)	0.7
Deep Sternal Infection	109 (3.3%)	45 (3.7%)	31 (2.5%)	0.10
Postoperative stroke	73 (2.1%)	27 (2.2%)	21 (1.7%)	0.4
Postoperative AF	643 (19%)	252 (20%)	207 (17%)	0.020

* Median (IQR); n (%); Mean (SD). ** Wilcoxon rank sum test; Pearson’s Chi-squared test; Fisher’s exact test. AF, Atrial Fibrillation; CABG, Coronary Artery Bypass Grafting; ICU, Intensive Care Unit; PCI, Percutaneous Coronary Intervention.

**Table 4:** Follow-up data.

		CTO/No CTO (Matched)		
Characteristic	Overall, N = 3424 *	CTO, N = 1232 *	No CTO, N = 1232 *	*p*-Value **
3-year mortality	277 (8.1%)	94 (7.6%)	67 (5.4%)	0.028
5-year mortality	417 (12%)	153 (12%)	100 (8.1%)	<0.001
10-year mortality	778 (23%)	302 (24%)	187 (15%)	<0.001
Coronary reinterventions after 3 years	228 (6.7%)	88 (7.2%)	86 (7.1%)	>0.9
Coronary reinterventions after 5 years	274 (8.1%)	106 (8.7%)	97 (8.0%)	0.5
Coronary reinterventions after 10 years	336 (9.9%)	143 (12%)	125 (10%)	0.2
Re CABG after 3 years	5 (0.1%)	2 (0.2%)	1 (<0.1%)	>0.9
Re CABG after 5 years	5 (0.1%)	2 (0.2%)	1 (<0.1%)	>0.9
Re CABG after 10 years	5 (0.1%)	2 (0.2%)	1 (<0.1%)	>0.9
PCI after 3 years	228 (6.7%)	88 (7.1%)	91 (7.4%)	0.8
PCI after 5 years	274 (8.1%)	107 (8.7%)	102 (8.3%)	0.7
PCI after 10 years	335 (9.9%)	138 (11%)	116 (9.4%)	0.14
Postoperative coronary angiography	616 (18%)	253 (21%)	185 (15%)	<0.001

* Median (IQR); n (%); Mean (SD). ** Wilcoxon rank sum test; Pearson’s Chi-squared test; Fisher’s exact test. CABG, Coronary Artery Bypass Grafting; PCI, Percutaneous Coronary Intervention.

**Table 5:** Cox multivariable regression (matched cohort).

Multivariable Cox Regression	HR	95%CI	*p*-Value
CTO	1.220	1.047–1.420	0.010
Age	1.055	1.044–1.066	<0.001
Hypertension	1.159	0.915–1.468	0.218
History of smoking	1.488	1.270–1.743	<0.001
History of malignancy	1.647	0.998–2.716	0.050
NYHA IV	1.936	1.051–3.567	0.034

CTO, Chronic total occlusion; NYHA, New York Heart Association.

## Data Availability

Data are unavailable due to privacy and ethical restrictions. The authors had full access to all the data in the study and take responsibility for the integrity of the data and the accuracy of the data analysis.

## Supplementary Materials

The following supporting information can be downloaded at https://www.mdpi.com/article/10.3390/jcdd12070243/s1. Statistical method (in-depth), characteristics/outcomes of the unmatched groups are found in the supplemental materials.
