# Supplementary material for: Impact of the Presence of Chronic Total Occlusions on the Survival of Patients Treated with Coronary Artery Bypass Grafting
Source: J Cardiovasc Dev Dis. 2025 Jun 25;12(7):243. doi: 10.3390/jcdd12070243 (PMC12295936; doi:10.3390/jcdd12070243)
Supplement: Supplementary file 1 [file jcdd-12-00243-s001.zip › jcdd-3549958-supplementary.pdf]

# Supplemental material

## Statistical analysis

Statistical analysis was performed using R Studio software (RStudio: Integrated Development for R. RStudio, Inc., Boston). Figures were created using the ggplot2 package (Version 3.5.0, ggplot2: Elegant Graphics for Data Analysis, Hadley Wickham). The Shapiro-Wilks test was used to assess the distribution of continuous data. Normally distributed data was expressed as +- standard deviation and compared using the unpaired T-test. Otherwise, the non-parametric Mann-Whitney-U test was used, and data was expressed as median and interquartile range. For categorical data, the Chi-square test was used. A P-value $< 0.05$ was deemed statistically significant.

To address confounders and strive for pseudo-randomization we performed propensity score matching before analyzing the data. To address missing data, we performed multiple imputations using the mice package (Multivariate Imputation by Chained Equation) using the CART algorithm for the following characteristics: Dyslipidaemia, smoking, hypertension, sex, diabetes mellitus, NYHA, coronary artery disease, chronic renal disease, left ventricular ejection fraction, as well as previous myocardial infarction(18). Matching was then performed using 1:1 optimal pair matching with propensity score distances estimated with logistic regression. Exact matching was used for LVEF, age, coronary artery disease, and dyslipidemia. This yielded a balanced dataset. Density plots, absolute standardized mean difference plots, and propensity score distribution plots can be found in the supplemental materials. To illustrate mortality and coronary reintervention Kaplan-Meier plots were used after matching. Additionally, multivariate, multivariable Cox-regression was used to estimate hazard ratio for the composite outcome over 10 years of follow-up. Proportional Hazards assumption was assessed using Schoenfeld-Residuals. Model selection was performed based on relative Akaike-Information Criterion (AIC) changes during repeat modelling to avoid overfitting.

**Table S1 Patient characteristics**

| Characteristics | Overall, N = 3,424[1] | CTO/No CTO (unmatched) CTO, N = 1,784[1] | No CTO,N = 1,640[1] | p-value[2] | CTO/No CTO (matched) CTO, N = 1,232[1] | No CTO,N = 1,232[1] | p-value[2] |
|---|---|---|---|---|---|---|---|
| Sex (female) | 532 (16%) | 255 (14%) | 277 (17%) | **0.036** | 172 (14%) | 186 (15%) | **0.4** |
| Age (years) | 68.0 (62.0, 74.0) | 68.0 (61.0, 74.0) | 69.0 (63.0, 75.0) | **<0.001** | 69.0 (63.0, 74.0) | 69.0 (63.0, 74.0) | **>0.9** |
| BMI | 27.4 (25.1, 30.1) | 27.7 (25.3, 30.4) | 26.8 (24.8, 29.4) | **<0.001** | 27.7 (25.4, 30.6) | 27.7 (25.4, 30.5) | **0.5** |
| Diabetes | 1,082 (32%) | 575 (33%) | 507 (32%) | **0.3** | 390 (32%) | 394 (32%) | **0.9** |
| Dyslipidemia | 2,728 (80%) | 1,484 (84%) | 1,244 (76%) | **<0.001** | 1,027 (83%) | 1,027 (83%) | **>0.9** |
| Hypertension | 2,844 (83%) | 1,510 (85%) | 1,334 (82%) | **0.019** | 1,053 (85%) | 1,046 (85%) | **0.7** |
| Arrhythmia | 294 (8.6%) | 148 (8.3%) | 146 (8.9%) | **0.5** | 103 (8.4%) | 110 (8.9%) | **0.6** |
| Smoking | 1,172 (37%) | 685 (41%) | 487 (33%) | **<0.001** | 469 (38%) | 436 (35%) | **0.2** |
| PAD | 447 (15%) | 238 (15%) | 209 (15%) | **0.5** | 167 (14%) | 169 (14%) | **>0.9** |
| COPD | 349 (10%) | 183 (10%) | 166 (10%) | **>0.9** | 122 (9.9%) | 113 (9.2%) | **0.5** |
| History of MI | 1,149 (34%) | 627 (35%) | 522 (32%) | **0.029** | 397 (32%) | 390 (32%) | **0.8** |
| History of stroke | 209 (6.1%) | 117 (6.6%) | 92 (5.6%) | **0.2** | 80 (6.5%) | 62 (5.0%) | **0.12** |
| Malignancy | 60 (1.9%) | 28 (1.7%) | 32 (2.2%) | **0.3** | 22 (1.8%) | 23 (1.9%) | **0.9** |
| NYHA | | | | **0.005** | | | **0.4** |
| 1 | 341 (11%) | 160 (11%) | 181 (12%) | | 154 (13%) | 164 (13%) | |
| 2 | 1,449 (47%) | 678 (45%) | 771 (49%) | | 595 (48%) | 613 (50%) | |
| 3 | 1,213 (39%) | 625 (41%) | 588 (37%) | | 470 (38%) | 448 (36%) | |
| 4 | 81 (2.6%) | 51 (3.4%) | 30 (1.9%) | | 13 (1.1%) | 7 (0.6%) | |
| Chronic renal failure | 97 (2.9%) | 64 (3.6%) | 33 (2.1%) | **0.010** | 18 (1.5%) | 14 (1.1%) | **0.5** |
| EuroScore II | 1.3 (0.9, 2.0) | 1.4 (0.9, 2.1) | 1.2 (0.9, 1.9) | **0.004** | 1.2 (0.9, 1.8) | 1.2 (0.9, 1.8) | **0.2** |
| Preop. LVEF < 30% | 88 (2.8%) | 56 (3.3%) | 32 (2.1%) | **0.032** | 5 (0.4%) | 5 (0.4%) | **>0.9** |
| Affected vessels | | | | **<0.001** | | | **>0.9** |
| 1 | 24 (1%) | 5 (0%) | 19 (1%) | | 1 (0%) | 1 (0%) | |
| 2 | 283 (8%) | 91 (5%) | 192 (12%) | | 53 (4%) | 53 (4%) | |
| 3 | 3,108 (91%) | 1,686 (95%) | 1,422 (87%) | | 1,178 (96%) | 1,178 (96%) | |

[1]Mean (SD); n (%); Median (IQR)

[2]Wilcoxon rank sum test; Pearson's Chi-squared test; Fisher's exact test

BMI, Body Mass Index; PAD, Peripheral Artery Disease; COPD, Chronic Obstructive Pulmonary Disease, MI, Myocardial Infarction;

## Table S2 Procedural characteristics

| | | CTO/No CTO (unmatched) | | | CTO/No CTO (matched) | | |
|---|---|---|---|---|---|---|---|
| **Characteristic** | **Overall**, N = 3,424[a] | **CTO**, N = 1,784[a] | **No CTO**, N = 1,640[a] | **p-value**[b] | **CTO**, N = 1,232[a] | **NO CTO**, N = 1,232[a] | **p-value**[b] |
| **No. of bypasses** | 3.1 (0.9) | 3.2 (0.9) | 3.0 (0.8) | <0.001 | 3.2 (0.9) | 3.1 (0.8) | <0.001 |
| **Complete revascularization** | 2,798 (91%) | 1,564 (91%) | 1,234 (92%) | 0.2 | 1,104 (90%) | 1,115 (91%) | 0.5 |
| **CPB (min)** | 90 (74, 107) | 91 (78, 107) | 88 (71, 106) | <0.001 | 90 (77, 105) | 89 (72, 107) | 0.10 |
| **Time of aortic cross-clamping (min)** | 69 (55, 84) | 69 (58, 83) | 68 (53, 85) | 0.073 | 69 (58, 81) | 69 (54, 85) | 0.8 |
| **Total time of surgery (min)** | 220 (190, 250) | 220 (195, 250) | 220 (190, 252) | 0.5 | 220 (190, 250) | 230 (190, 256) | 0.068 |
| **OPCAB** | 316 (9.2%) | 174 (9.7%) | 142 (8.7%) | 0.3 | 23 (1.9%) | 38 (3.1%) | 0.052 |

[a1]Mean (SD); n (%); Median (IQR)

[b]Wilcoxon rank sum test; Pearson's Chi-squared test; Fisher's exact test

CPB, cardiopulmonary bypass, OPCAB, off-pump coronary artery bypass grafting

**Table S3 30-day Outcomes**

| Characteristic | Overall, N = 3,424[a] | CTO/No CTO (unmatched) | | | CTO/No CTO (matched) | | |
|---|---|---|---|---|---|---|---|
| | | CTO, N = 1,784[a] | No CTO, N = 1,640[a] | p-value[b] | CTO, N = 1,232[a] | No CTO, N = 1,232[a] | p-value[b] |
| **30-day mortality** | 59 (1.7%) | 33 (1.8%) | 26 (1.6%) | 0.6 | 18 (1.5%) | 16 (1.3%) | 0.7 |
| **Coronary reinterventions after 30 days** | 95 (2.8%) | 55 (3.1%) | 40 (2.5%) | 0.3 | 37 (3.0%) | 35 (2.9%) | 0.8 |
| **Surgical coronary reintervention after 30 days** | 4 (0.1%) | 2 (0.1%) | 2 (0.1%) | >0.9 | 1 (<0.1%) | 1 (<0.1%) | >0.9 |
| **Percutaneous coronary reintervention after 30 days** | 95 (2.8%) | 55 (3.1%) | 40 (2.5%) | 0.3 | 37 (3.0%) | 36 (2.9%) | >0.9 |
| **Length of hospital stay (days)** | 14.0 (11.0, 17.0) | 14.0 (11.0, 18.0) | 13.0 (10.0, 15.0) | <0.001 | 14.0 (11.0, 17.0) | 13.0 (11.0, 16.0) | <0.001 |
| **Length of ICU stay (days)** | 1.0 (0.9, 1.9) | 1.0 (0.9, 1.9) | 1.0 (0.9, 1.7) | 0.021 | 0.9 (0.9, 1.8) | 0.9 (0.8, 1.6) | 0.10 |
| **Postoperative permanent Pacemaker** | 49 (1.4%) | 26 (1.5%) | 23 (1.4%) | >0.9 | 18 (1.5%) | 17 (1.4%) | 0.9 |
| **Postoperative Dialysis** | 113 (3.3%) | 61 (3.4%) | 52 (3.2%) | 0.7 | 26 (2.1%) | 29 (2.4%) | 0.7 |
| **Deep Sternal Infection** | 109 (3.3%) | 66 (3.8%) | 43 (2.7%) | 0.083 | 45 (3.7%) | 31 (2.5%) | 0.10 |
| **Postoperative stroke** | 73 (2.1%) | 46 (2.6%) | 27 (1.7%) | 0.062 | 27 (2.2%) | 21 (1.7%) | 0.4 |
| **Postoperative atrial fibrillation** | 643 (19%) | 361 (20%) | 282 (17%) | 0.027 | 252 (20%) | 207 (17%) | 0.020 |
| **Postoperative LVEF < 30 %** | 63 (2.0%) | 38 (2.4%) | 25 (1.7%) | 0.2 | 12 (1.0%) | 10 (0.8%) | 0.7 |

[a]Median (IQR); n (%); Mean (SD)

[b]Wilcoxon rank sum test; Pearson's Chi-squared test; Fisher's exact test

ICU, Intensive Care Unit; LVEF, Left Ventricular Ejection Fraction

**Table S4 Follow-up data**

| Characteristic | Overall, N = 3,424[a] | CTO/ No CTO (unmatched) | | p-value[b] | CTO/ No CTO (matched) | | p-value[b] |
|---|---|---|---|---|---|---|---|
| | | CTO, N = 1,784[a] | No CTO, N = 1,640[a] | | CTO, N = 1,232[a] | No CTO, N = 1,232[a] | |
| **3-year mortality** | 277 (8.1%) | 163 (9.1%) | 114 (7.0%) | 0.020 | 94 (7.6%) | 67 (5.4%) | 0.028 |
| **5-year mortality** | 417 (12%) | 254 (14%) | 163 (10.0%) | <0.001 | 153 (12%) | 100 (8.1%) | <0.001 |
| **10-year mortality** | 778 (23%) | 484 (27%) | 294 (18%) | <0.001 | 302 (24%) | 187 (15%) | <0.001 |
| **Coronary reinterventions after 3 years** | 228 (6.7%) | 125 (7.1%) | 103 (6.4%) | 0.4 | 88 (7.2%) | 86 (7.1%) | >0.9 |
| **Coronary reinterventions after 5 years** | 274 (8.1%) | 156 (8.8%) | 118 (7.3%) | 0.10 | 106 (8.7%) | 97 (8.0%) | 0.5 |
| **Coronary reinterventions after 10 years** | 336 (9.9%) | 199 (11%) | 137 (8.5%) | 0.006 | 143 (12%) | 125 (10%) | 0.2 |
| **Surgical coronary reintervention after 3 years** | 5 (0.1%) | 3 (0.2%) | 2 (0.1%) | >0.9 | 2 (0.2%) | 1 (<0.1%) | >0.9 |
| **Surgical coronary reintervention after 5 years** | 5 (0.1%) | 3 (0.2%) | 2 (0.1%) | >0.9 | 2 (0.2%) | 1 (<0.1%) | >0.9 |
| **Surgical coronary reintervention after 10 years** | 5 (0.1%) | 3 (0.2%) | 2 (0.1%) | >0.9 | 2 (0.2%) | 1 (<0.1%) | >0.9 |
| **PCI after 3 years** | 228 (6.7%) | 125 (7.1%) | 103 (6.3%) | 0.4 | 88 (7.1%) | 91 (7.4%) | 0.8 |
| **PCI after 5 years** | 274 (8.1%) | 156 (8.8%) | 118 (7.2%) | 0.10 | 107 (8.7%) | 102 (8.3%) | 0.7 |
| **PCI after 10 years** | 335 (9.9%) | 198 (11%) | 137 (8.4%) | 0.007 | 138 (11%) | 116 (9.4%) | 0.14 |
| **Postoperative coronary angiography** | 616 (18%) | 374 (21%) | 242 (15%) | <0.001 | 253 (21%) | 185 (15%) | <0.001 |
| **Composite endpoint** | 1034(31%) | 622(35%) | 412(25%) | <0.001 | 404(32.7%) | 292(23.6%) | <0.001 |

[a]Median (IQR); n (%); Mean (SD)

[b]Wilcoxon rank sum test; Pearson's Chi-squared test; Fisher's exact test

PCI, Percutaneous coronary reintervention

**Table S5 Cox Multivariable Regression**

| Multivariable Cox Regression | HR | 95%CI | p-value |
|---|---|---|---|
| **CTO** | 1.220 | 1.047 – 1.420 | 0.010 |
| **Age** | 1.055 | 1.044 – 1.066 | <0.001 |
| **Hypertension** | 1.159 | 0.915 – 1.468 | 0.218 |
| **History of smoking** | 1.488 | 1.270 – 1.743 | <0.001 |
| **History of malignancy** | 1.647 | 0.998 – 2.716 | 0.050 |
| **NYHA IV** | 1.936 | 1.051 – 3.567 | 0.034 |
| **Preoperative CK-MB** | 0.999 | 0.996 – 1.003 | 0.915 |

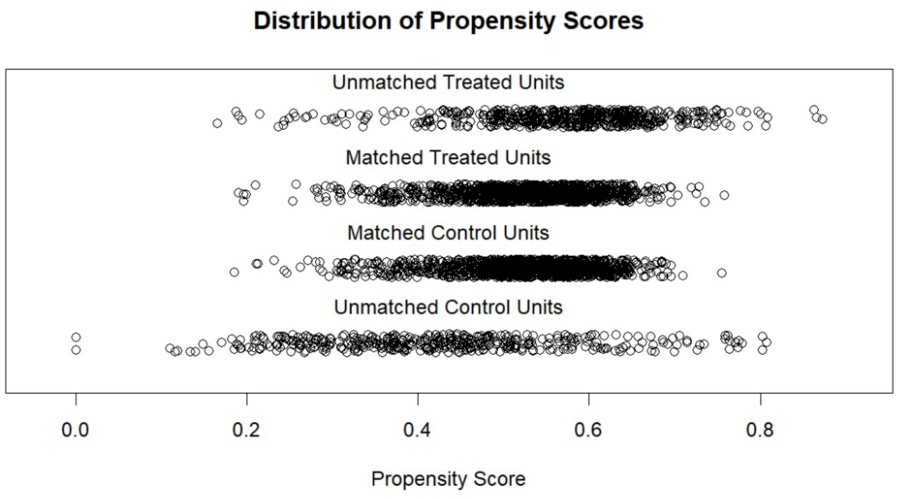
Distribution of Propensity Scores
Unmatched Treated Units
Matched Treated Units
Matched Control Units
Unmatched Control Units
0.0
0.2
0.4
0.6
0.8
Propensity Score

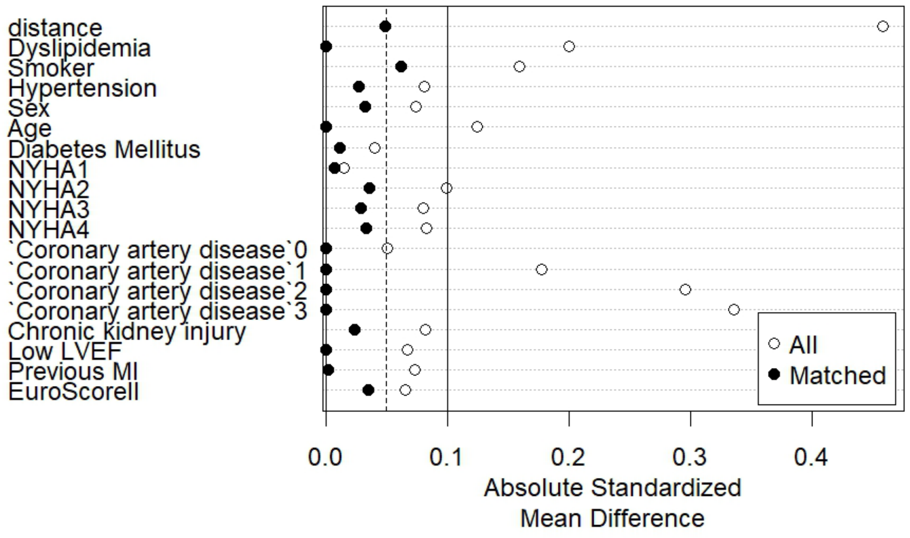
distance
Dyslipidemia
Smoker
Hypertension
Sex
Age
Diabetes Mellitus
NYHA1
NYHA2
NYHA3
NYHA4
`Coronary artery disease`0
`Coronary artery disease`1
`Coronary artery disease`2
`Coronary artery disease`3
Chronic kidney injury
Low LVEF
Previous MI
EuroScoreII
All
Matched
0.0
0.1
0.2
0.3
0.4
Absolute Standardized
Mean Difference

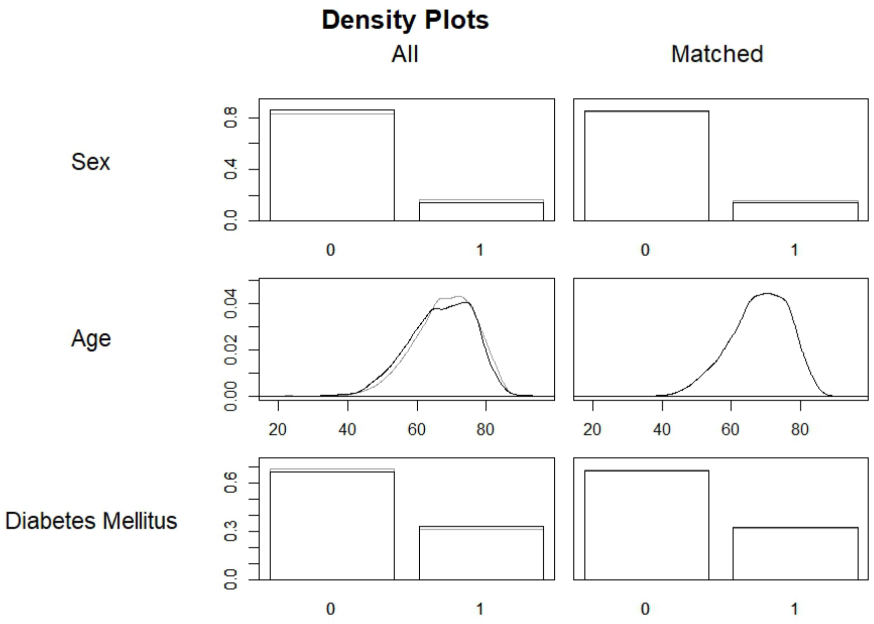
Density Plots
All
Matched
Sex
0.0
0.4
0.8
0
1
Age
0.00
0.02
0.04
20
40
60
80
Diabetes Mellitus
0.0
0.3
0.6
0
1

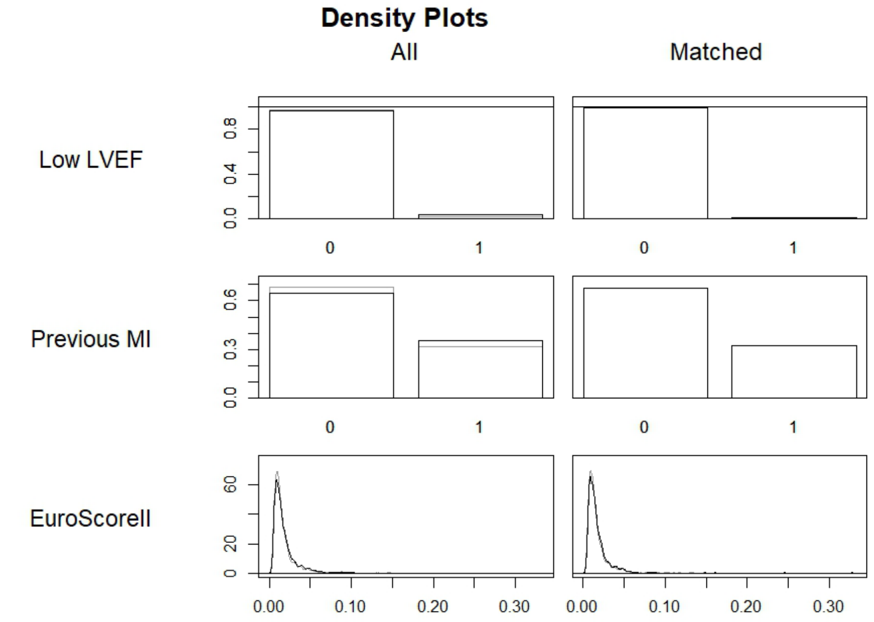
Density Plots
All
Matched
Low LVEF
0.0
0.4
0.8
0
1
Previous MI
0.0
0.3
0.6
0
1
EuroScoreII
0
20
60
0.00
0.10
0.20
0.30

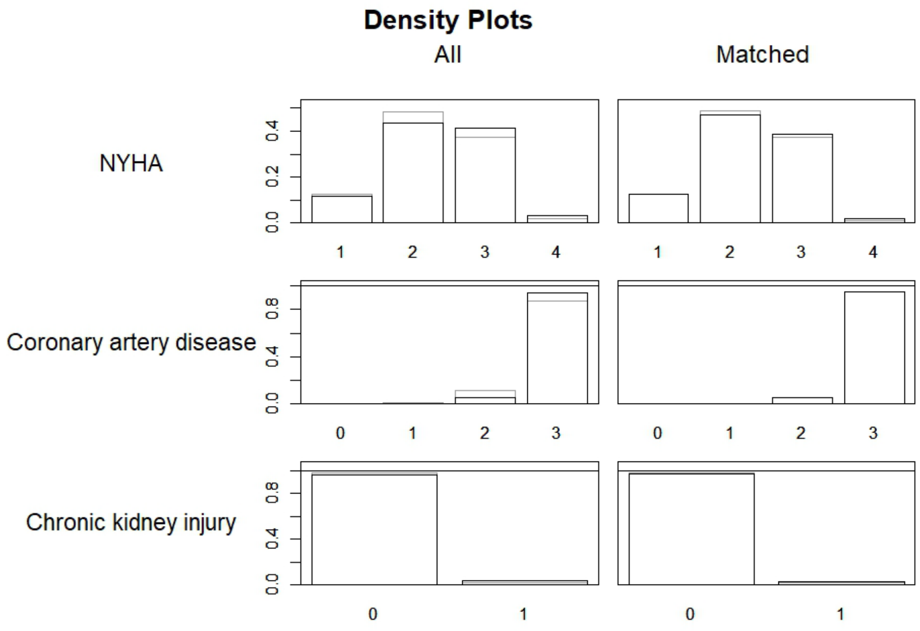
Density Plots
All
Matched
NYHA
Coronary artery disease
Chronic kidney injury
0.0
0.2
0.4
0.8
1
2
3
4
0
